# Distribution and orientation of nerve fibers and myelin assembly in a brain section retrieved by small-angle neutron scattering

**DOI:** 10.1038/s41598-021-92995-2

**Published:** 2021-08-27

**Authors:** Santanu Maiti, Henrich Frielinghaus, David Gräßel, Martin Dulle, Markus Axer, Stephan Förster

**Affiliations:** 1 Jülich Centre of Neutron Science (JCNS-1/IBI-8), Forschungszentrum Jülich GmbH, 52425 Jülich, Germany; 2 Institute of Neuroscience and Medicine (INM-1), Forschungszentrum Jülich GmbH, 52425 Jülich, Germany; 3 Jülich Centre for Neutron Science at Heinz Maier-Leibnitz Zentrum (JCNS-MLZ), Forschungszentrum Jülich GmbH, 85748 Garching, Germany; 4 Institute of Physical Chemistry, RWTH Aachen University, 52074 Aachen, Germany

**Keywords:** Imaging, Characterization and analytical techniques

## Abstract

The structural connectivity of the brain has been addressed by various imaging techniques such as diffusion weighted magnetic resonance imaging (DWMRI) or specific microscopic approaches based on histological staining or label-free using polarized light (e.g., three-dimensional Polarized Light Imaging (3D-PLI), Optical Coherence Tomography (OCT)). These methods are sensitive to different properties of the fiber enwrapping myelin sheaths i.e. the distribution of myelin basic protein (histology), the apparent diffusion coefficient of water molecules restricted in their movements by the myelin sheath (DWMRI), and the birefringence of the oriented myelin lipid bilayers (3D-PLI, OCT). We show that the orientation and distribution of nerve fibers as well as myelin in thin brain sections can be determined using scanning small angle neutron scattering (sSANS). Neutrons are scattered from the fiber assembly causing anisotropic diffuse small-angle scattering and Bragg peaks related to the highly ordered periodic myelin multilayer structure. The scattering anisotropy, intensity, and angular position of the Bragg peaks can be mapped across the entire brain section. This enables mapping of the fiber and myelin distribution and their orientation in a thin brain section, which was validated by 3D-PLI. The experiments became possible by optimizing the neutron beam collimation to highest flux and enhancing the myelin contrast by deuteration. This method is very sensitive to small microstructures of biological tissue and can directly extract information on the average fiber orientation and even myelin membrane thickness. The present results pave the way toward bio-imaging for detecting structural aberrations causing neurological diseases in future.

## Introduction

The brain serves as the center of our nervous system and consists of gray and white matter. White matter accounts for around 40% of the human brain^1^. It is composed of fiber bundles of mostly myelinated axons that allow rapid transmission of action potentials to distant brain areas including the spinal cord. The myelin sheath surrounding the axon electrically insulates the axonal membrane to allow fast saltatory signal transduction. Injury to the myelinated white matter or diseases leading to the loss of myelination severely deteriorate brain and body functions such as in multiple sclerosis (MS), causing serious neurodegenerative diseases and psychological disorders^2,3^.

Therefore, there is great interest in the development of methods to study the myelinated axon fiber distribution in the brain. The investigation of the fiber distribution has received increasing attention in recent years, because the three-dimensional fiber network structure of the brain reveals the connectivity of the axonal network (connectome) that is necessary to understand dysfunctions of the brain^4^. Hence, methods that provide accurate local information on the myelin structure and orientation have become important tools in brain research. Established methods to determine local fiber structure and orientation are diffusion weighted MRI (DWMRI), 3D Polarized Light Imaging (3D-PLI), Optical Coherence Tomography (OCT) and confocal laser scanning microscopy (CLSM) to name only a few.

DWMRI provides orientation-based/tractography-modelled descriptions of nerve fiber pathways in the entire brain at the millimeter scale in case of in vivo measurements^5^, down to a few hundred microns in the post mortem case^6^. It probes the diffusion length of water molecules as a function of the spatial direction^5–8^. Due to the concentric wrapping of the myelin sheath around the axon, water molecules within the myelin sheath can diffuse over large distances parallel to the axon^9^, whereas long-distance diffusion normal to the axon direction is strongly hindered by multiple myelin bilayer membranes^10,11^. Recent MRI studies demonstrated the ability to extract the myelin distribution across the human brain^12^. Note, the measured MRI/DWMRI signal is sensitive to myelin, but also to other tissue components and microstructures, which makes a clear disentanglement of the myelin difficult.

3D-PLI probes the birefringence originating from the optical anisotropy of the rod-shaped lipid molecules constituting the myelin bilayer membrane^13–16^, Because of the molecular optical anisotropy, there is a difference between the refractive index parallel to the bilayer membrane compared to the value perpendicular to the membrane^17,18^. In recent years, 3D-PLI demonstrated in various applications to different species its benefit in determining the local fiber axis direction at a few micrometer resolution^19^. Optical coherence tomography (OCT) delivers depth-resolved cross-sectional images of microstructures in the biological tissue with micrometer scale resolution^20^. Using birefringence properties of myelin, this technique can be used to differentiate white matter from gray and to determine fiber orientations in ex-vivo brain^21–23^. Confocal laser scanning microscopy (CLSM) can be applied to image fluorescently stained thin brain section^24^. Myelin staining can provide 3D information about the local nerve fiber distribution by 3D volume reconstruction of a series of brain sections. CLSM achieves 0.2 μm in-plane resolution^25^. Myelin immunostaining can further reveal differences in the myelination pattern between healthy and diseased brains^26^.

As each method has specific limitations due to the lack of spatial resolution, sensitivity to non-axonal signal contributions and uncertainty concerning signal pre-factors, comparisons between these methods have been conducted to verify the 3D fiber orientation distributions. This has been done e.g. with confocal microscopy vs. DWMRI^27^ or DWMRI vs. PLI^28^. Recently, small angle X-ray scattering (SAXS) has been introduced as a further method to probe the myelin structure as well as its 3D orientational distribution^29^. SAXS provides the Bragg diffraction peaks resulting from the periodic concentric arrangement of the lipid bilayers in the myelin sheath^30–33^. As the azimuthal position of the Bragg peaks on the detector depends on the 3D-orientation of the myelinated axons, a series of data with different inclination angles allows to quantitatively reconstruct the 3D orientational distribution^32,34–37^. It was possible to quantitatively correlate the 3D fiber orientation distribution function (fODF) with DWMRI.

A method that can provide valuable complementary information on the structure and local orientation of myelin sheaths is scanning small angle neutron scattering (sSANS). Neutron scattering is isotope-sensitive such that deuteration can be used to selectively enhance the scattering intensity of biomolecules such as lipids and proteins^38^. Compared to X-ray scattering, the neutron has no negative water/lipid contrast regions such that both, at low- and intermediate scattering angles high scattering intensities can be detected due to the lipid-based microstructural assembly^39,40^ So far neutrons have been employed on the brain to characterize the average structural information of the gray matter, and the white matter and to investigate the dynamics of water molecules in a brain^41–43^. However,  neutrons have not yet been used for determining the structure and orientation of myelin or fibers in an entire brain section.

We show for the first time that neutron scattering can be used to characterize the myelin distribution and orientation in thin brain sections. By adjusting the wavelength spectrum and beam divergence of an intense neutron beam at a research reactor facility we were able to map the neutron scattering intensity over a complete rodent brain section at a resolution of 1 mm^2^, which is comparable to DWMRI. We found that it is possible to resolve the Bragg peaks of the myelin sheath revealing the in-plane orientation of myelinated nerve fibers. The results were validated by 3D-PLI. We furthermore found that the anisotropy of the low-angle scattering profiles is directly related to the average nerve fiber orientation. With the advent of future powerful neutron spallation source the determination of structural and orientational information of the myelin sheath in a brain at higher spatial resolution will become possible.

## Results

### 3D-PLI fiber orientation map

**Figure 1:**
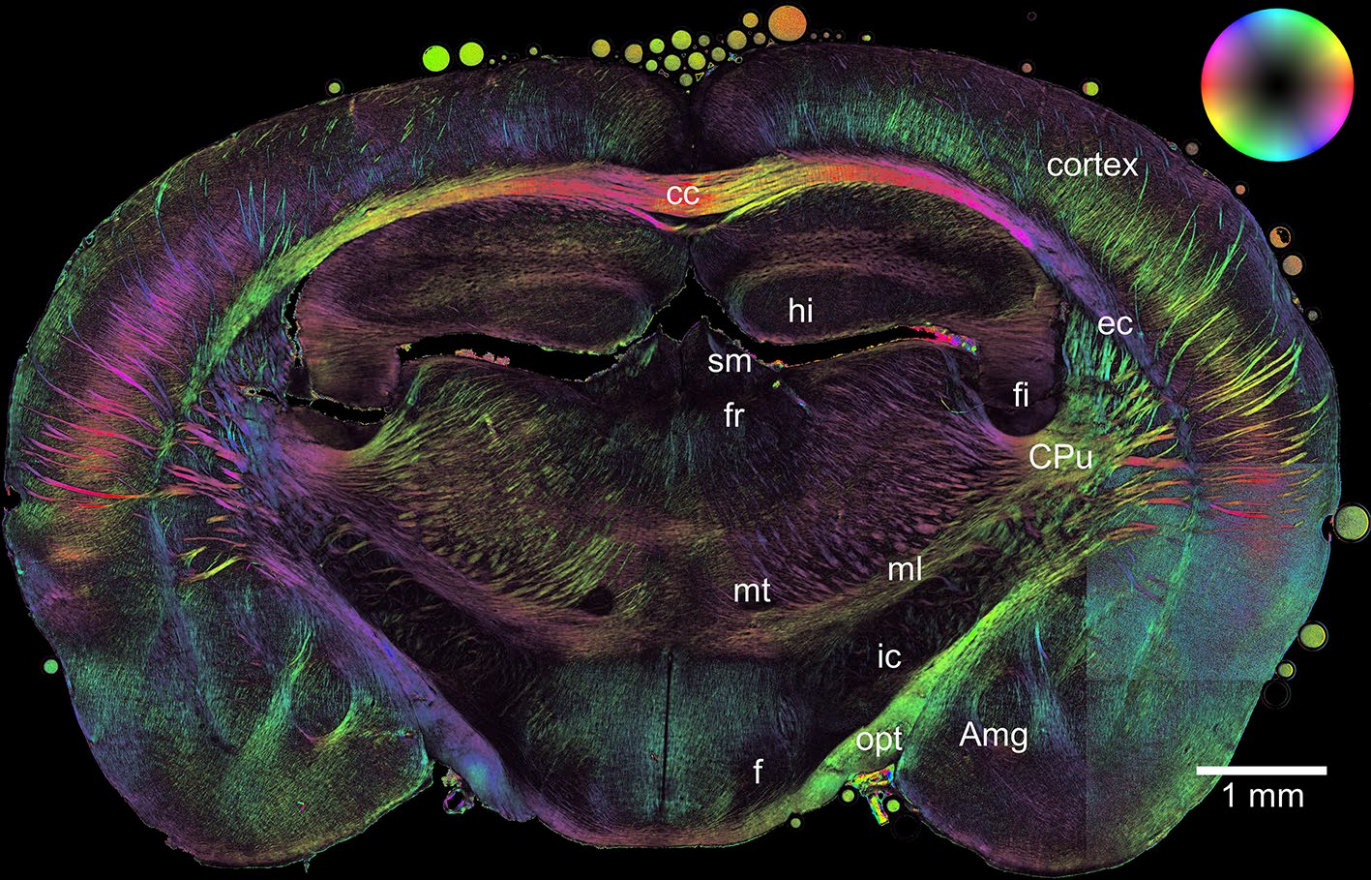
The 3D fiber orientation map (FOM) of a reeler mouse brain section. Anatomical parts of the section are labeled for reference; *cc* corpus callosum, *hi* hippocampus, *sm* stria medullaris, *fr* fasciculus retroflexus, *fi* fimbria hippocampi, *CPu* caudate putamen, *mt* mammilothalamic tract, *ml* medial lemniscus, *ic* internal capsule, *opt* optic tract, *Amg* amygdala, *f* fornix, *ec* external capsule. Color bubble: The color-coding corresponds to the orientation of the fibers.

Figure 1 displays the three-dimensional orientation and distribution map of the nerve fibers i.e. the FOM of a coronal reeler mouse brain section, generated by means of 3D-PLI microscopy measurement. The image provides information about the spatial orientation of the nerve fibers in the entire brain section at a spatial resolution of 1.3 μm. The fiber orientations are coded by colors in the HSV-color space. The in-plane projection of the fiber orientation is coded by hue, the out-of-plane component is coded by saturation and value (see color bubble). Red corresponds to horizontal directions, cyan to vertical ones. Color saturation and brightness are attenuated in steep fibers. The anatomical parts of the brain section are labeled in the FOM for reference, each of them showing a different fiber architecture (i.e., fiber bundles, tracts, and individual fibers). In this study, we compare the structural information obtained from the scanning SANS measurements with 3D-PLI.

### Scanning SANS

**Figure 2:**
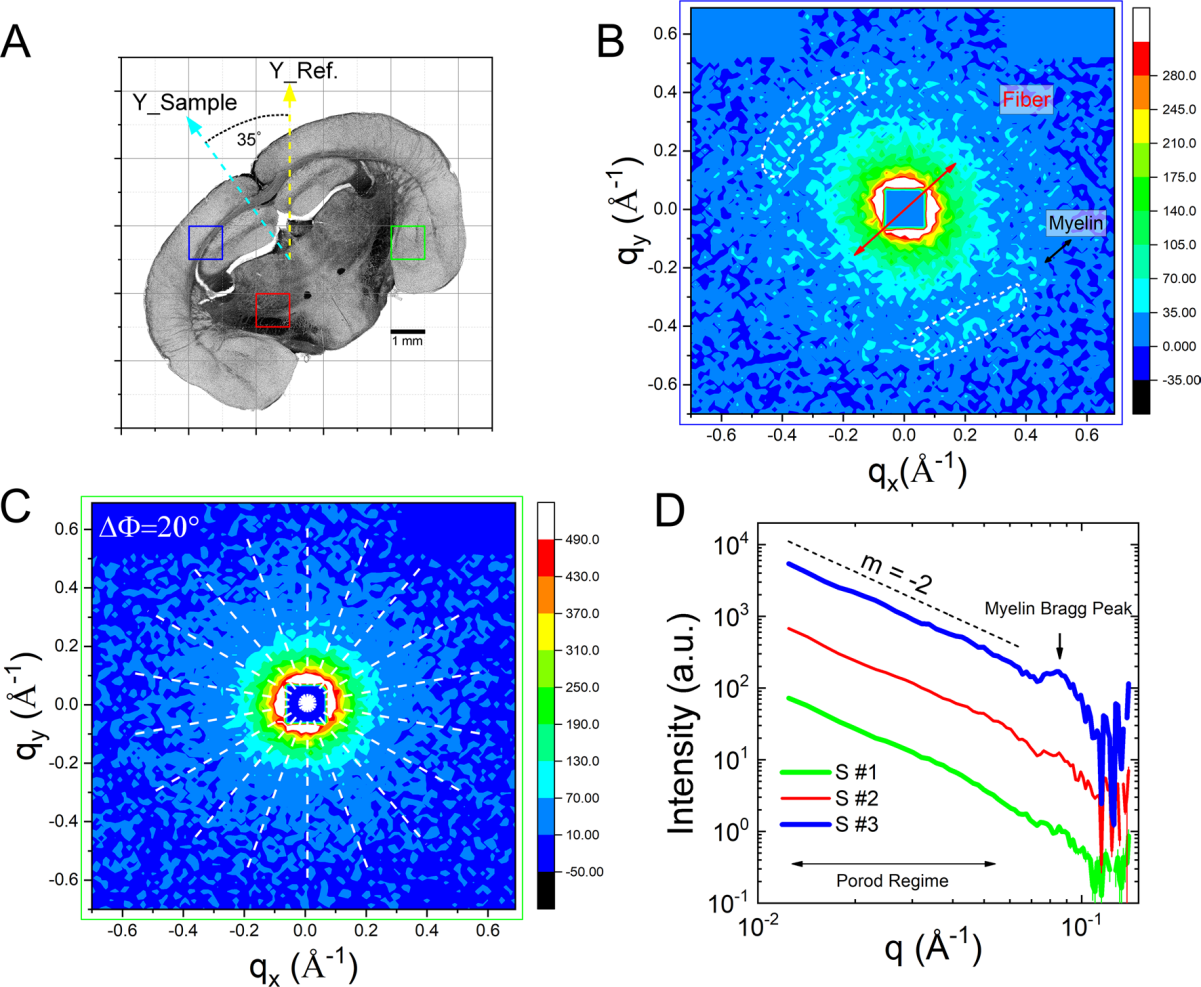
(**A**) A polarization-independent light transmission microscopy image of the coronal section of the reeler mouse brain. Few scanning areas are labeled (square boxes of different color) for reference. (**B**) A typical SANS pattern in *q-*space (*q*_*x*_*, q*_*y*_) obtained at the cc region (blue square box). There is a pronounced low-q scattering with a notable anisotropy indicated by the red arrow, and a pair of DS arcs with an anisotropic diffraction intensity indicated by the black arrows corresponding to the collateral alignment of the bilayer membranes in the myelin sheaths inside the callosal commissure. (**C**) A typical SANS pattern in *q-*space obtained in a subcortical nucleus (green square box), showing isotropic scattering at low-*q*, without having any DS arc. (**D**) Azimuthally averaged SANS-intensity as a function of the scattering vector ($\left| q \right| = \sqrt {q_{x}^{2} + q_{y}^{2} }$) at three different positions in the brain section (square boxes in (**A**) with similar color). The data are shifted vertically by a factor of 10 for clarity. The scattering intensity varies with a characteristic *q*^−2^ power law at low-*q*. The myelin Bragg peak is observable near 0.083 A^−1^ and their intensity drastically depend on the position inside the brain section undergoing the scan.

Figure 2A displays a polarization-independent bright-field image of the same section, presented in line with the rectangular scanning geometry. The entire sample has been scanned by a square millimeter neutron beam for the corresponding scattering patterns. Figure 2B shows a typical 2D SANS pattern as obtained from a scan on the white matter region of the brain section (‘cc’, red square box). It has two distinct regimes—namely the Porod regime (near the direct beam) and a regime of two azimuthally symmetric scattering arcs/peaks in the periphery, marked by white dotted ellipses.  The signal in the Porod regime reflects forward scattering events by the microstructures present in the section. Interestingly, the 2D pattern in the Porod regime has  a shape of an anisotropic ellipse. The signal anisotropy relates to an oriented distribution of structures with a strongly anisotropic shape. The scanning point is located in the neighboring region of corpus callosum (‘cc’), which is made of in-plane nerve fibers. Therefore, this scattering feature can be attributed to the oriented assembly of nerve fibers/bundles. The direction of the corresponding fibers can be estimated accordingly and denoted by a red arrow in the pattern. Apart from the scattering in the Porod regime, two scattering arcs are clearly visible at two symmetrically opposite positions across the direct beam. They are attributed to the second order myelin Bragg peaks (n =2) located on the Debye–Scherrer (DS) ring at* q*_max_ = 0.083 Å^−1^^41^. In real space, the position of the peak corresponds to a length scale (t) of 15.2 nm  $$\left( {t = {\raise0.7ex\hbox{${2\pi}n$}\!\mathord{\left/ {\vphantom {{2\pi } {q_{{max}} }}}\right.\kern-\nulldelimiterspace} \!\lower0.7ex\hbox{${q_{{max}} }$}}} \right)$$., which is the average periodicity of lipid membrane in a myelin sheath. Moreover, the azimuthal position of the scattering spots/arcs on the DS-ring depends on the predominant orientation of the myelin sheath of fibers. In real space, the orientation of the myelin sheath/fiber is perpendicular to the orientation of Bragg peaks as labeled by a typical black arrow.

Figure 2C is a typical scattering pattern obtained from a scanning position in a subcortical nucleus (green square box). This pattern has an isotropic distribution at low-*q* with respect to the direct beam (*q* = 0). The isotropic scattering pattern corresponds to isotropic homogeneously distributed assemblies of micro-structural objects in the region. Importantly, there is no DS ring/arc in this scattering signal. The absence of a characteristic DS ring in the pattern can be attributed to the absence of a significant quantity of myelin in that location.

 During scanning across the brain section, we observed a continuous change in the shape of the scattering anisotropy either at Porod or Bragg regime, which corresponds to the orientational change of the local tissue micro-structures. To evaluate the signal anisotropy for every scan position, each pattern has been sampled into 9 diametric segments (white dotted lines in Fig. 2C). The scattering intensity is integrated over the dihedral angle covered by each segment as a function of the momentum transfer *q*. The result is finally weighted by the corresponding number of detector elements to determine the center symmetry of the pattern.

Figure 2D shows typical radial profiles as a function of the momentum transfer vector *q*, collected at three entirely different parts (as marked in Fig. 2A) of the section. The profiles are the resultant integrated intensity over all the sensors of the detector. There are two distinct regimes discriminable in these radial profiles. The low-*q* forward scattering part of each curve (Δ*q*_*Porod*_: 0.02–0.06 Å^−1^) with a scattering intensity according to a *q*^−2^ power law. The slope of the curves (m =  − 2) is characteristic of the form factor of 2D structures such as bilayer membrane or locally flat disk-like structures occurring in the myelin. The radial width of the Bragg diffraction peaks reflects the thickness distribution of the lipid multilayers. The scattering intensity of the Bragg peak varies over the different scanning positions on the brain section (Fig. 2D) which corresponds to the degree of myelination (amount of lipid in the fibers). We estimated the myelin Bragg peak position (*q*_max_) from the scattering profiles and mapped it across the section (Fig. S3). A slight difference in *q*_max_-position has been observed and that could be attributed to bilayer periodicity variation in the myelin sheath over the brain section.

### Fiber distribution and orientation map

**Figure 3:**
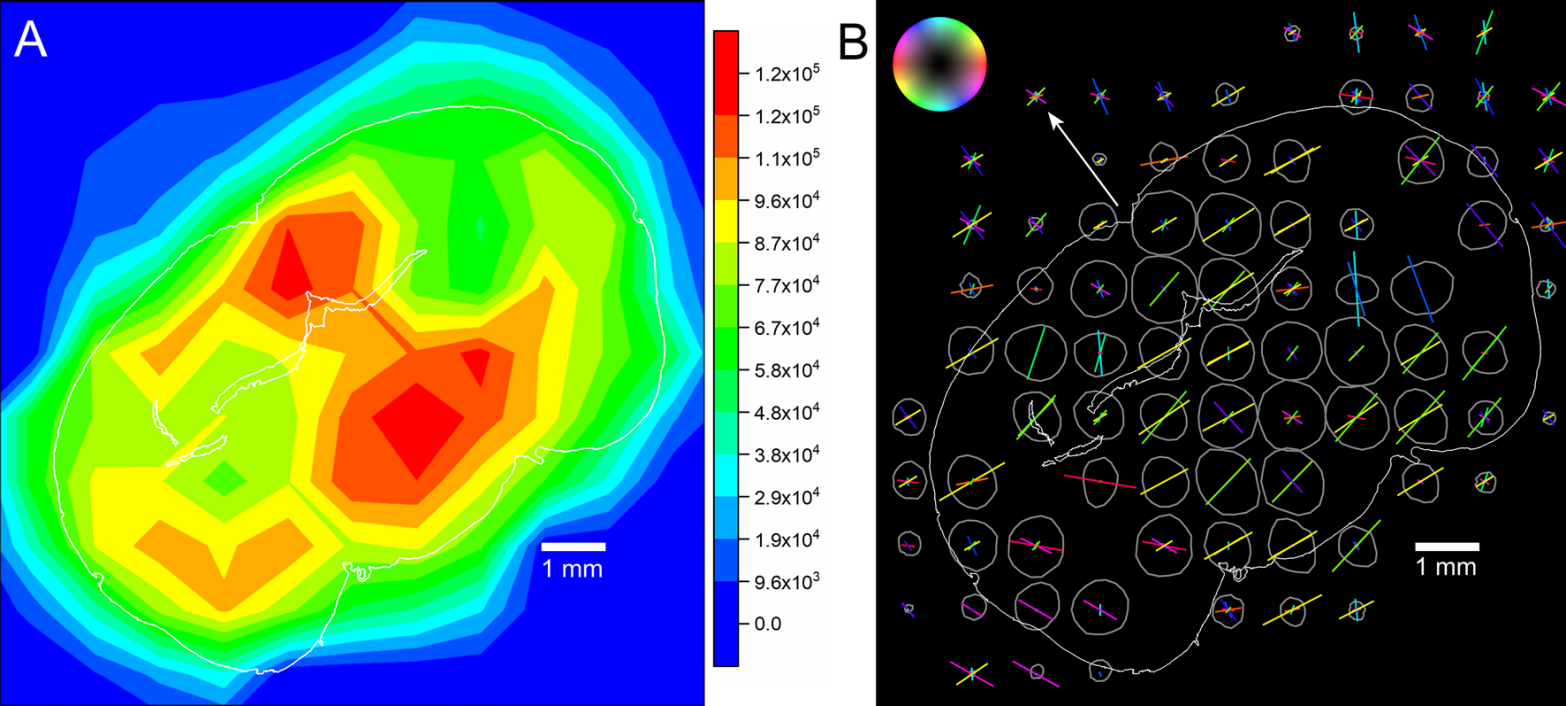
(**A**) 2D contour map of the integrated SANS intensity in the Porod regime or dark-field map. The color bar on the right side quantifies the scattering intensity. The obtained map is divided into following symmetrical regions. α: strong intensity region (red, orange), located in the caudal interhemispheric area; β: intermediate intensity region (faded orange and yellow), extending to both hemispheres; γ: the cranial interhemispheric area dominated by the callosal commissure, elongated towards the left hemisphere; ζ and η: low intensity regions (green), distributed over the cortical periphery and subcortical nuclei of each hemisphere, respectively. (**B**) Scattering intensity distribution profile and orientation map of the nerve fibers, as obtained from SANS intensity in the Porod regime. The colors of the bars correspond to the in-plane direction of the fibers according to the color legend on top left, while the length of the bars reflect the peak prominence in the scattering intensity profiles (gray ellipsoids). The white peripheral line indicates the silhouette of the brain section. The white arrow points to the top side of the brain.

Figure 3A shows the contour map of Porod intensity or dark-field map over the entire brain section. The contours of the scattering intensity levels depend on the geometric interpolation due to coarse resolution (1 mm). Depending upon scatting intensity distribution, we divided the map into 5 different regions. The highest scattering intensity occurs in the lower-central region (red, ~ 1 × 1 mm^2^) and it falls off evenly in all neighboring areas (orange/yellow, size ~ 3 × 2 mm^2^). The entire region is labeled as α-region. The shoulder-like symmetric regions (orange/yellow) with a 20% drop of intensity is designated as β-region. The other region with strong intensity at the upper-central part of the section (red/orange/yellow, ~ 2 × 2 mm^2^) is demonstrated as γ-region. A low scattering intensity (green) is evidenced in the rest of the map. The two-symmetric regions with half of intensity as seen at α-region or less (green) are indexed as η-region. The other low scattering part is located along the cortical periphery (~ 1.5 mm, green) is indicated as ζ-region. Eventually, the scattering intensity fades out towards the edge of the sample.

Figure 3B displays the scattering intensity distribution profile (gray fingerprints) and orientation of nerve fibers in each tile as obtained from Porod analysis. The colored bars represent the directions of the fibers dominating the respective area. On a square millimeter area, however we expect mixtures of fibers in almost every tile. The intensity distribution peaks in the Porod regime are much broader than the Bragg peaks at the periphery; hence the peaks near the beam center are not separable from each other. The central regions (α and γ) show not only a higher scattering intensity but also a higher anisotropy compared to the cortex regions (ζ). High scattering intensity peaks inside the tissue merge into average directions (e.g. ml, mt) with exception of the central part of the α-region, which is characterized by balanced vertical and horizontal fiber contributions producing two distinct scattering peaks. In the cortex, the structures parallel to the brain surface are pronounced, with an exception for the most lateral segment, where the cortex is interfused by radial fiber bundles. This type of malformation is typical for the brain of reeler mouse.

### Myelin distribution and orientation map

**Figure 4:**
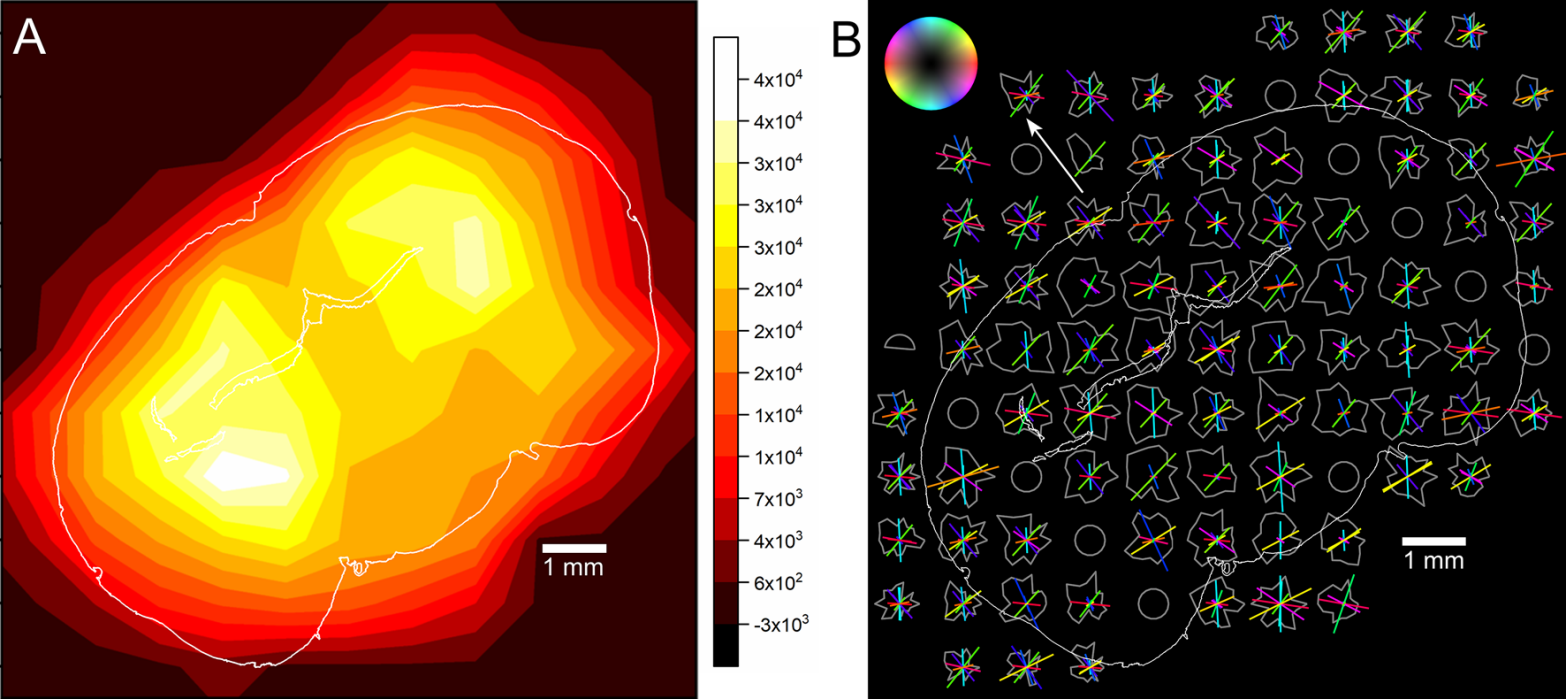
(**A**) 2D contour map of myelin Bragg peak intensity or MBPI map. The extracted map is divided into following symmetrical regions. Π: high scattering intensity area (white), located at symmetrically opposite position and almost at the middle of each hemisphere; Σ: extended Σ-region with an intermediate scattering intensity (white + yellow); *∆*: relatively low scattering area (faded yellow), at the bridging area of two Σ-regions; Ω: lowest scattering intensity region (orange), distributed mostly over the gray matter. (**B**) Bragg peak intensity distribution profile and orientation map of the myelin sheath/fibers as obtained from the SANS intensity in the Bragg regime. The colors of the bars correspond to the in-plane direction of the myelin sheath/fibers according to the color legend on top left, while the length of the bars reflect the prominence of the Bragg peaks in the scattering intensity profiles (gray fingerprints). The white peripheral line indicates the silhouette of the brain section. The white arrow points to the top side of the brain. 10 measurements inside the tissue (gray circles) were skipped due to time constraints.

Figure 4A shows the resultant intensity distribution from the myelin Bragg peak intensity (MBPI) across the entire brain section. The entire MBPI map has been divided into the four specific regions. The high scattering intensity region (white) which is located at the symmetrically opposite position and at almost the middle of each hemisphere is indexed as Π-region. The extended region with slightly lower scattering intensity (white + yellow) across the Π-region in each lobe is designated as Σ-region. The region with intermediate intensity distribution in the bridging area of two Σ-regions (connecting area of hemispheres) is marked as Δ-region. The rest of region with lower Bragg intensity along periphery (orange) is indexed as Ω-region. The intensity of Bragg peak primarily depends on the amount of myelin existing in the brain tissue. Hence, the stronger scattering regions like Π- and Σ-regions contain a significant amount of myelin in comparison to the rest. The strong scattering regions (white, Π** -**region) at two symmetrically identical positions implies that the myelin is concentrated highly in a small area at each hemisphere. The uniformly distributed scattering intensity in the Δ-region with a lower intensity (intermediate-range, yellow) implies to have a moderate amount of myelin present in this region. The weak myelin scattering intensity over the rest of the MBPI map indicates the existence of a smaller amount of myelin in the rest of the brain section.

Figure 4B shows the intensity distribution profiles along the DS ring (gray fingerprints) and the fiber/myelin directions extracted from the Bragg peaks as colored bars. The position of the Bragg peaks are at the periphery of each SANS pattern. DS arcs well-separated from the beam center allow for a more detailed sampling in comparison to the Porod regime, however still considering a coarse azimuthal resolution of 20°. The number of peaks detected in the profiles is higher than in the beam center and varies between 4 up to 6. For this reason, the location of the peaks alone is not sufficient to characterize the fiber architecture, hence we evaluated the prominence and even the width of each peak to determine their impact (see Fig. S4, includes the peak width). High prominence (long bars) mainly occurs in regions dominated by one or two flat fiber directions, whereas lower prominence (short bars) indicates steeper or multiple fiber directions. The gray matter (Ω) is generally crowded by many short bars, whereas some principal directions are getting more prominent in the white matter regions (Σ, Δ). An exception occurs in the most lateral cortex regions showing again prominent radial fiber bundles as seen already in the Porod regime. Main fiber tracts in the white matter (cc, ml, mt, opt) are retraced by the Bragg peaks, however not always with the maximum prominence. The tissue borders of the ventricles below the hippocampus seem to induce horizontally aligned scattering peaks producing artificial vertical direction components throughout their whole neighborhood (blue magenta bars). A similar behavior i.e. a very prominent centrifugal direction component is noticeable all around the brain border. For direct comparison of histology and SANS, a combined figure (Figs. 1, 3B and 4B in side-by-side) is available in the Supplementary Information as Fig. S5.

### 3D-PLI maps

**Figure 5:**
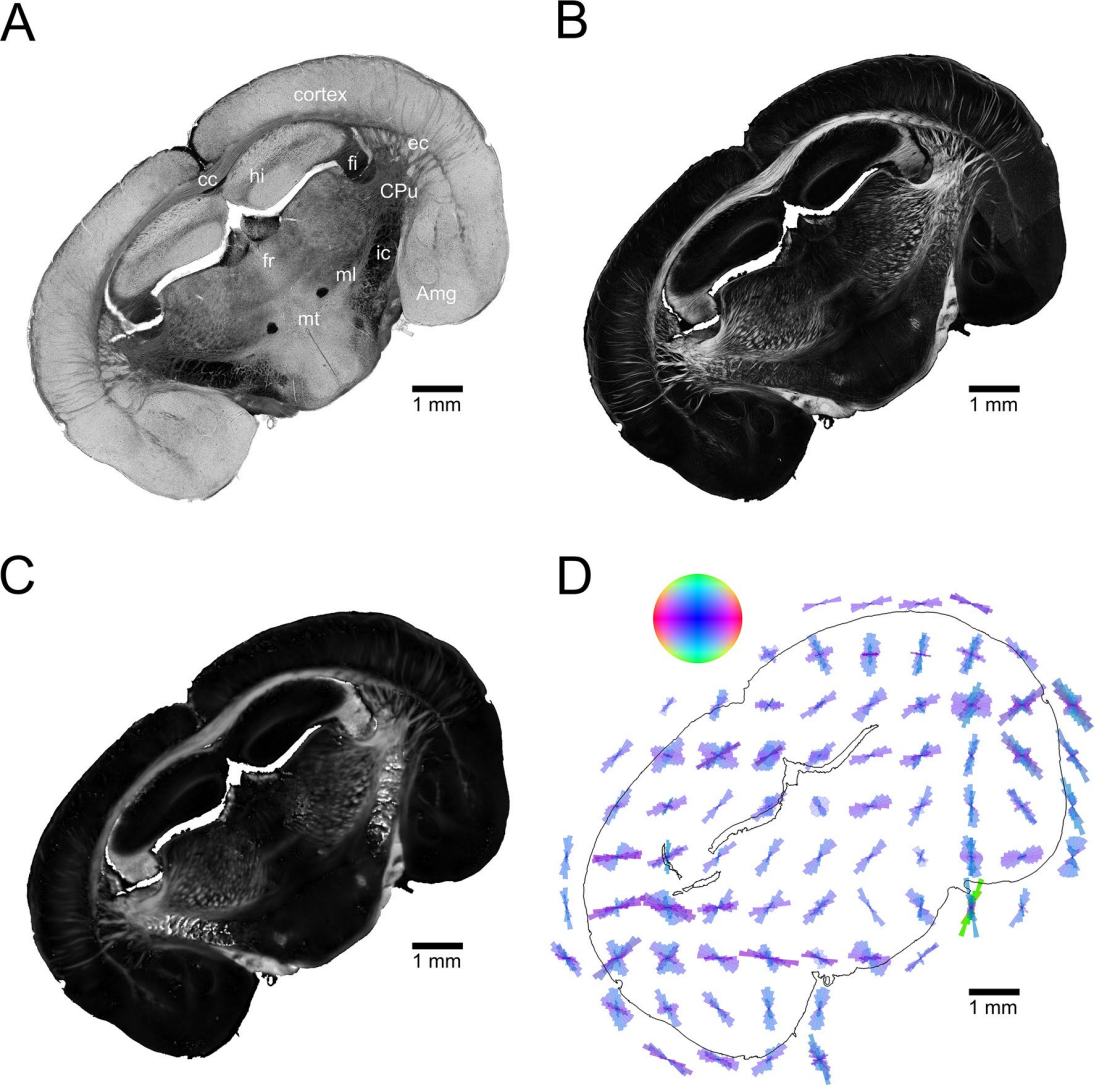
Different modalities of the brain section as obtained from the 3D-PLI microscopy measurement. (**A**) Normalized transmittance image. (**B**) Retardation image. (**C**) Relative thickness map, representing the myelin density. (**D**) Top down view of the spherical 3D-histograms of the vector data with a spatial resolution of 1 mm × 1 mm as obtained by gathering each mm^2^ tile (769 × 769 vector objects) of the high resolution (1.3 μm) 3D-PLI vector array into a single histogram (cf. Fig. 1). The peripheral line (white) is the contour line of the registered brain section.  The color of the 3D-histograms corresponds to the color code shown at the top left.

Figures 5A,B show the 3D-PLI normalized transmittance and retardation image of the same section, respectively. In general, the transmittance is reproducibly higher in the gray matter as compared to the white matter. However, the transmittance varies on its anatomical properties like myelin density or fiber inclination. Myelinated fiber tracts appear dark. The dense fiber bundles in the cortex are specific for the reeler mouse and are not present in commonly investigated laboratory mouse strains. The retardation ‘δ’ is high in regions where the myelinated fibers have low inclination running within the plane of sectioning (α ~ 0°, e.g. cc). Low retardation in a white matter region either reflects fiber structures pointing steeply out of the sectioning plane (α ~ 90°, e.g. ‘fr’) or nearly 90° fiber crossings (e.g. ‘ec’). Moreover, we determined the relative thickness (t_rel_) values from the retardation values (Fig. 5C) which reflects the orientation-independent birefringence of the entire mouse brain section, which is highly correlated to the myelin density.

Figure 5D shows the PLI vector data condensed to 3D-histograms representing the fiber orientations of PLI at SANS resolution (1 mm) to allow for direct comparison with the peaks found in the neutron scattering profiles (cf. Fig. 3B, 4B). Here, we avoided the trap of 3D-PLI vector averaging, yielding false oblique results for orthogonal contributions, choosing a spherical histogram solution showing the total variety of the fiber orientations in each 1 mm^2^ voxel. In order to maintain the visibility of steeper fibers the RGB color space is preferred here instead of HSV. The dominant shade of blue can be attributed to the noise from non-birefringent objects located primarily in the gray matter.

### Comparing the results of sSANS and 3D-PLI

**Figure 6:**
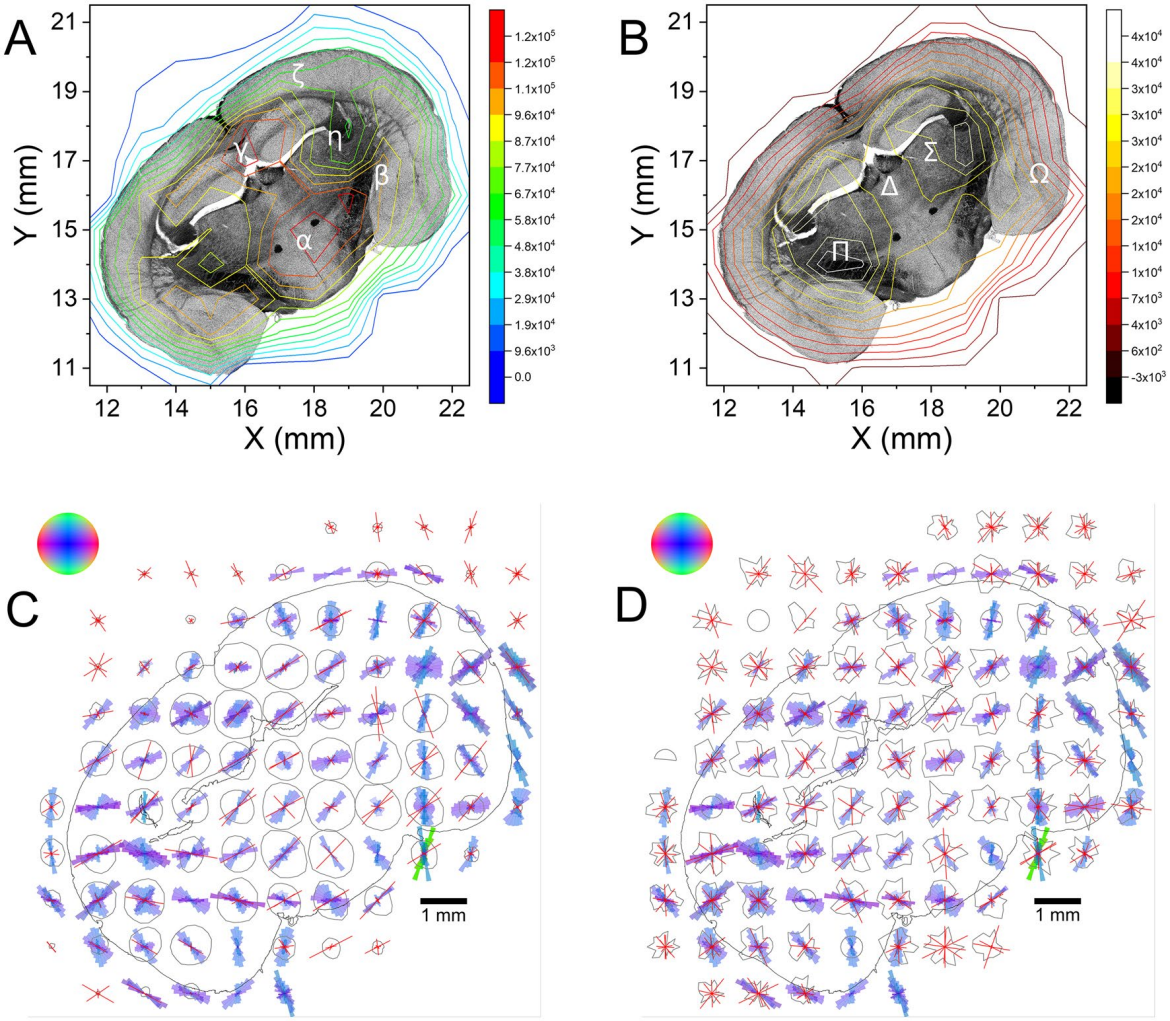
(**A**) The contour lines of the SANS dark-field map in the Porod regime, registered on top of the 3D-PLI transmittance image. α (red + orange lines): lower-central diencephalon zone; β (orange + yellow lines): Amg, opt and ic; γ (red + orange lines): area of cc and hi; ζ (green lines near periphery): cortex. η (green lines): fi, ec and CPu. (**B**) The contour lines of the MBPI map, registered on top of the 3D-PLI transmittance image. Π (white line): fi and CPu; Σ (yellow line): entire η-region of dark-field map, e.g. area of fi, ec and CPu; *∆* (bridging area): cc, sm, fr and mt; Ω (red lines): cortex, Amg and opt. (**C**)–(**D**) Superimposed map of combined azimuthal intensity profiles (gray fingerprints) and the extracted peaks (red bars) in Porod regime and in Bragg regime on top of the 3D histograms of cumulative 769 × 769 PLI vector data in each square millimeter (cf. Fig 5D). The lengths of the bars indicate the peak prominence i.e. the single peak amplitude divided by the amplitude of the whole 360° intensity profile. The standard RGB color legend (red=horizontal, green=vertical, blue=perpendicular) on the top left reflects the fiber directions in the PLI 3D vector histograms. The location of the brain section is delineated by the black borderline.

Figures 6A,B illustrate a qualitative comparison of two different SANS maps (dark-field and MBPI) with the 3D-PLI image. An accurate registration of SANS contour lines (Figs. 4A, 5A) on top of 3D-PLI transmittance assists to identify the anatomical parts of the brain section. The ζ-region (green lines along periphery) of dark-field map relates to the Ω-region (red lines) of MBPI map. This region corresponds to the entire cortex along the cortical periphery (see Fig. 1). The scattering intensities of these regions are relatively low. The SANS anisotropy of dark field map is rather low in the upper half of cortex but increases remarkably in the lower half, strictly parallel to the brain surface. The Bragg peak azimuth (Fig. 6D) at the upper part, coarsely reflects the radially oriented reeler mouse specific fibers of the cortex, whereas at the lower half, it resembles the anisotropy of the SANS pattern. However, the fiber orientation histograms of 3D-PLI clearly exhibit the orthogonal fiber architecture inside the cortex. Therefore, the fiber architecture is not retraced by the scattering anisotropy especially at the lower half of cortex.

The η-region (green circular lines) of dark-field map extends to the Σ-region of the MBPI map. These regions relate to ‘fi’, ‘CPu’ and ‘ec’, where the tracts are massively ordered (Fig. 1) and the region at the middle of ‘hi’, ‘ic’, and ‘ec’, where the fibers run randomly. These regions show surprisingly low scattering intensity in dark-field map (η), whereas, the analogous areas (Σ, Π) in the MBPI map display a very high scattering intensity. In principle, the MBPI map reflects the myelin density of the specimen i.e. dark areas in the transmittance map and bright areas in the retardation map. Therefore, it is not surprising to see the scattering intensity maxima in Σ and Π, which are dominated by massively myelinated fibers like in ‘fi’ and ‘CPu’. However, the low intensity distribution in dark-field map (η) means that SANS patterns has significantly low scattering in Porod regime (at low-*q*). Moreover, Σ and Π are flanked by steep fiber bundles clearly visible in transmittance map of ‘fi’, and even reflected perfectly by the Bragg peak azimuths in the vicinity, which are good candidates for absorbing neutrons at small scattering angles.

The β-region (yellow lines) of the dark-field map covers the extended Σ-region of the MBPI map. These regions correspond to ‘Amg’, ‘opt’ and ‘ic’. It shows a distribution of scattering intensity of intermediate range in dark-field (yellow lines) as well as in MBPI map (yellow lines). This region is confined medially by steep fibers of the internal capsule (dark retardation and dark transmittance) and laterally by transversal fibers of the optic tract (bright retardation and dark transmittance). The fiber direction of these two tracts is reasonably reflected by the directions of the SANS patterns. The transversal prevalence of the fiber directions due to the vertical arrangement of the Bragg peak azimuths in the left hemisphere on the left side might be due to flat tracts penetrating the much steeper internal capsule from medial to lateral.

The γ, α-regions (red lines at the top and the bottom, respectively) and their interconnection in the dark-field corresponds to upper, lower and middle portion of Δ-region (yellow lines) of the MBPI map. These regions cover the entire central portion of the section. These regions exhibit high scattering intensity in dark-field and overall low to intermediate intensity in MBPI map. Here, a discrepancy between the SANS scattering modalities is visible along the interhemispheric fissure. While MBPI is maximal in the center directly below the third ventricle and drops to the vertical periphery, the dark-field intensity shows a reverse gradient. Steep fibers near ‘sm’ and ‘fr’ could be the reason for low scattering intensity in the dark-field, while MBPI keeps at its level. On the other hand, MBPI drops significantly at ‘hi’, while the dark-field scattering intensities reach their maximum. These regions are characterized by a mixture of gray matter (bright transmittance) pervaded by loose volatile fiber architecture, reflected by a weak anisotropy in the dark-field scattering.

To verify the directionality of the fiber architecture by two different methods, we compared the peaks detected in the azimuthal SANS intensity profiles with PLI in each tile. Figure 6C shows an overlay of the scattering profile of the Porod regime with the 3D-histograms of the PLI-Data already presented in Fig. 5D. In particular, the extracted SANS and PLI show a high conformity in the strong scattering regions (large gray drops in the center). The highest compliance of the SANS in the Porod regime with PLI however occurs along the margins of the brain section originating from myelinated nerve fiber projections above the cortical plate running along the surface of the mouse cortex, even amplified by neutral background contributions reducing the heterogeneity of scattering in these tiles. In deeper layers of the gray matter, the fiber directions of SANS and PLI also resemble remarkably. The white matter skeleton (cc, mt, ml, opt) is retraced by SANS satisfactorily. In the optic tract of the right hemisphere however discrepancy massively increases due to a horizontal bias in the SANS vector orientation governing the whole area (f, ic, Amg).

We further compared the fiber orientation, as derived from the SANS Bragg peak azimuth with the spherical 3D fiber histograms. In a nerve fiber, the myelin sheaths are wrapped coaxially around the axon of the fibers. Therefore, the orientation of myelin sheaths are collinear to the the direction of the fibers (refer Figs. S1, S2). Figure 6D shows the superimposed plot of the Bragg peak azimuth in the SANS pattern with the PLI histograms. The Bragg Peaks are not necessarily arranged in pairwise opposition especially for inclined fibers aligned close to the neutron flight direction. Therefore, depending on their inclination (content of blue in the PLI 3D-histograms, cf. color legend) several couples of deviating directions taken from non-opposite single peaks might belong to the same fiber tract. Especially in the cortex this behavior is obvious for blue (strongly inclined) fiber contributions, reproduced by symmetric pairs instead of single bars, slightly deviating from the actual direction to the left and to the right. A general reconstruction of the fiber inclination from associated peak pairs however fails due to the very coarse azimuth resolution (20°). Nevertheless, apart from some Bragg peak artifacts near the ventricles and the borderline of the brain already mentioned in Fig. 4B, the majority of the fiber directions derived from the Bragg peaks matches the directionality of the PLI 3D-histograms, all the more taking into account the peak width (see Fig. S4). For a direct comparison of histology, SANS and PLI, a combined figure (Figs. 1, 6C and 6D in side-by-side) is available in the Supplementary Information as Fig. S6.

## Discussion

To identify the neuronal network of a brain, we have considered a brain section of a reeler-mouse as a model system. It is a special type of mutated mouse shows the characteristic reeling gait^44^. This nature is usually characterized by the profound underdevelopment of the brain where the neurons are produced normally but are frequently organized into random manner in the central nervous system. The reason to choose the reeler mouse model, in particular, is the occurrence of appropriately sized fibers even in the cortex providing a narrow vicinity of gray matter and strongly oriented white matter with solid fiber tracts which is normally not present that much in normal brains. Therefore, the structural information of such a model could assist to find the link towards brain development.

Nerve fibers in a brain have been studied since 1930s by traditional histology^27,45,46^, electron microscopy^47^, dMRI^48^, synchrotron micro-CT^49,50^ and polarized light microscopy^13,17^. In last few decades, various X-ray scattering techniques have been used to investigate the myelin sheath in nerve fibers^30,31,33,51–53^. X-rays measure the electron density contrast (number of electrons present in the atoms) of the sample^54^ and determine distinct structural features of intermodal myelin: the lipid polar head group layers, the hydrocarbon tails of the bilayer and the aqueous spaces^55^. The scattering power of X-rays for the three basic elements (O, C and N) present in biological samples is very similar, therefore, X-rays cannot distinguish between these elements and determine an average of a heterogeneous membrane along the plane of the bilayer. As a result, the complete structural determination requires additional biochemical or structural correlations.

Neutron scattering provides a complementary insight for a better understanding about the distribution and orientation of myelin/fibers in a brain section. In earlier studies, neutron has been employed to investigate some selected region or specific fibers of the brain and to investigate the dynamics of water in the brain^41,42^. It is worth mentioning that for those studies, they used either thick sample (in order of mm) or scanned them with a larger neutron beam (> 10 mm). As a result, they always observed concentric isotropic rings in the scattering patterns by neutron diffraction. Instead of using a thick-sample, a thin-section of brain (~ 60 μm) has been employed in the present study. Furthermore, the scattering contrast has been enhanced by substitution of H_2_O by D_2_O isomorphously^43^. In contrast to X-rays, neutrons are scattered by the atomic nuclei and the scattering depends on their scattering length of an element. The scattering lengths often vary significantly between isotopes of the same element. By replacing hydrogen (_1_H or H) atom with the deuterium (_2_H or D) in the brain samples, the incoherent scattering from hydrogen is suppressed significantly^56^. This deuteration procedure potentially enhances the coherent scattering from the sample via enhancing scattering length, which allows us to estimate the structural organization of myelin/fibers in the brain section more precisely.

Polarized light has been developed into a powerful tool as 3D-PLI for determining the nerve fiber (i.e. myelinated axon) orientations and their distribution (FOD) across the brain^14^. It measures the transmittance of the polarized light and analyzes the optical anisotropy (birefringence), caused by the alignment and composition of the myelin sheath wrapped around the nerve fibers. Here, the transmittance signal of the light is highly anisotropic as the section thickness (60 µm) is much higher than the spatial resolution of the camera (1.3 µm), which often leads to partial volume effects in a single voxel. In case of voxels comprising fibers running in multiple directions the FOD cannot be determined reliably as the average birefringence signal drops significantly. Moreover, for the anatomical regions with densely packed nerve fibers, neither the inclination angles nor the in-plane distribution of the nerve fibers can be disentangled by 3D-PLI.

On the other hand, SANS can directly deliver structural information on the molecular level (lipid bilayer) and provide the directional and spatial distribution of the myelin as well as the nerve fibers across the brain section. The heterogeneous fiber architecture of the reeler mouse model of a convenient dimension is reasonably fit for a neutron scattering environment. Therefore, in the current study, we tend to image of myelinated nerve tissue on a resolution scale of about 100 microns up to one millimeter independent of the species. It can serve as another potential method to quantify the amount of fiber or myelin, existing across the entire brain section.

Conventional SANS measurements of fibrous brain samples provide usually only 2D information about the underlying fiber architecture. While sSANS is a non-destructive technique to map biological samples, it has its limitations. Limitations in neutron flux, even at high-power reactors, presently limit the resolution of scanning SANS to 1 mm. Long exposure time is required to have a good quality of data, which is not ideal for a mapping. The future availability of high-flux neutron spallation sources will improve the resolution by using smaller beam-sizes and will provide high-resolution maps at shorter exposure times and improved data quality to derive microstructure information (quantifying extract amount of myelin, thickness variation of myelin sheath, structural parameter of the lipid bilayers) on smaller scales. Nevertheless, the micro-scale resolution as delivered by 3D-PLI will remain out of reach for neutron scattering.

In conclusion, here, we describe a novel methodology to map the nerve fiber and myelin density, structure, and orientation in a brain section, based on the sSANS. With the adequate settings of the beam (size and flux), we map the fiber and myelin scattering intensity to detect the fiber-rich and myelin-rich regions across a complete brain section, together with their local lamellar packing. The 2D anisotropic scattering patterns allow to extract the in-plane orientation of nerve fibers and myelin assembly in a brain section. The obtained structural information at a spatial resolution of 1 mm is comparable to the resolution of in-vivo dMRI. This opens up the possibility to validate the structures, obtained through various tractograpy or connectivity methods. The results of this study could pave the way toward imaging of myelin of the laboratory animals. Additional 3D structural information can be deduced by rotating the sample with respect to the beam and by selective deuteration of the sample. In the future, the availability of high-flux spallation sources will enhance spatial resolution to the micrometer range, making the study of microstructure in biological tissues generally feasible.

## Methods

### Mouse model

The brain of an adult male reeler-mouse is used as a model system for this study^44^. The brain of the mouse was surgically extracted from the skull directly after death and preserved in 4% buffered formaldehyde (BFA) solution for a few days. Finally, the brain was frozen by immersion in isopentane at − 50 °C for a few hours. The animal procedures were approved by the institutional animal welfare committee at the Research Centre Jülich GmbH, Germany, and were in accordance with European Union guidelines for the use and care of laboratory animals.

### Brain section preparation

The frozen brain was completely sectioned along the coronal plane at a thickness of 60 µm using a cryostat microtome (Leica Microsystems, Germany) at a temperature of − 30 °C. A single section was selected from the rear part of the mouse brain to maximize the variety of tissue in the sample (width: 10 mm, height: 6 mm). The section was mounted on cooled glass slides. Then 20% glycerin was added, and the section was covered by another coverslip to carry out the 3D-PLI microscopy measurement. Afterwards, the brain section was transferred onto a sapphire glass window, treated with D_2_O (deuterated) solvent for an hour to enhance the neutron contrast, and covered by another sapphire glass plate of 1 mm thickness. The section was mounted on a sapphire glass plate, covered by another sapphire plate. Before sealing the sample, a solution of 20% glycerin was added to prevent it from drying out before the start of scattering measurements. The sandwiched sample was implanted near the center of circular aperture on a metallic frame (Fig. S7). After mounting the sample, the section was measured with the neutron beam a few days later in the SANS beamline.

### SANS measurement

**Figure 7:**
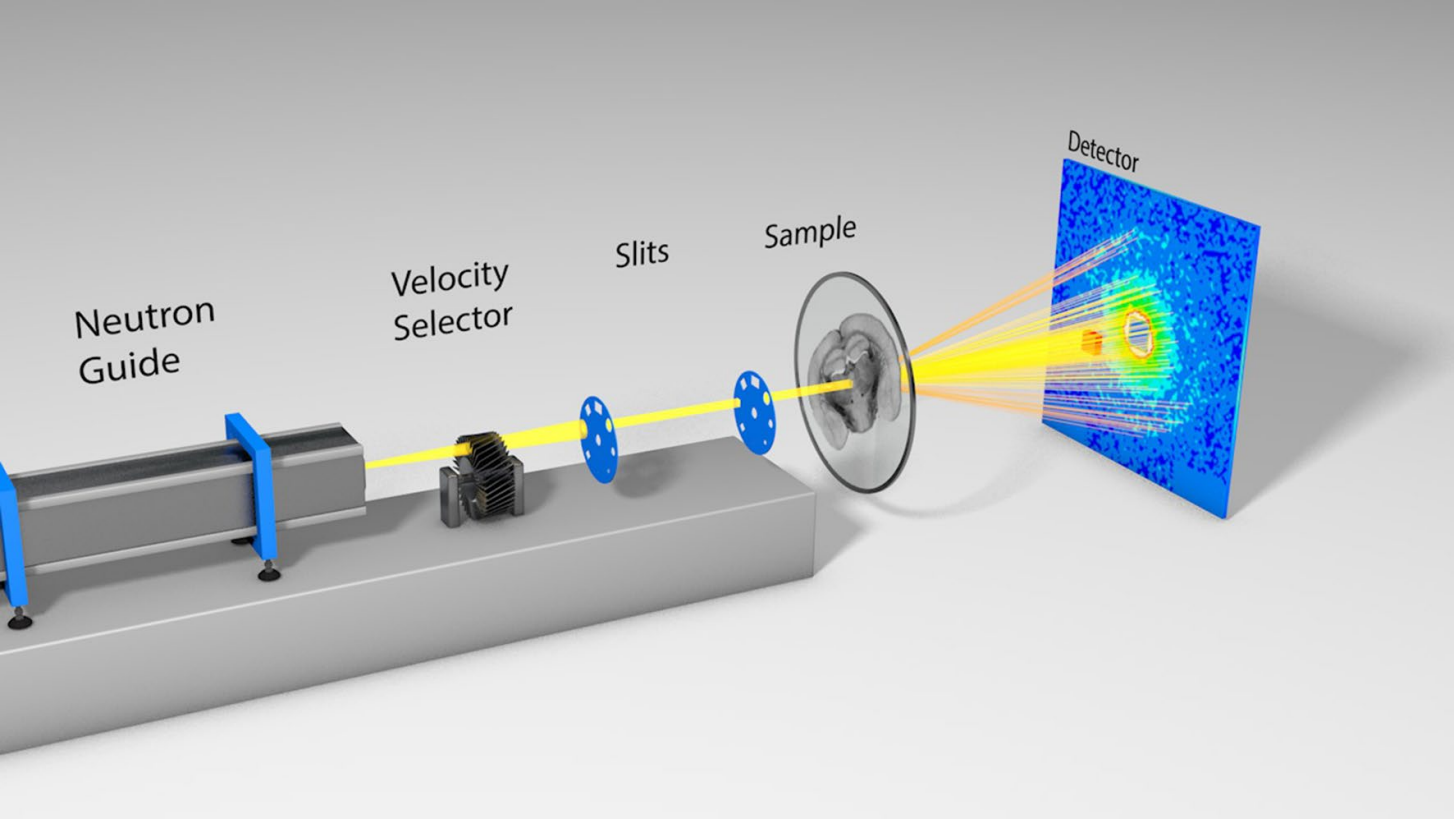
Scheme of the scanning SANS geometry to study the mouse brain section. During measurement, the transversal orientation of the brain section had a counter-clockwise offset of 35° with respect to the horizontal alignment of the laboratory frame.

SANS measurements were carried out at the small-angle scattering diffractometer (KWS-1) beamline at MLZ in Garching, Germany, which is operated by the Jülich Centre for Neutron Science (JCNS)^57^. A schematic of the scanning SANS geometry to study the brain section is presented in Fig. 7. Two pairs of collimating slits were used in the vertical and horizontal direction to get a square sized beam of 1 × 1 mm^2^. This is the minimum possible beam size to obtain sufficient neutron flux in the spray cone. To maximize neutron flux at the sample position maintaining sufficient resolution and to resolve the Bragg peaks in the scattering patterns, the mean wavelength was tuned to λ = 0.5 nm at the maximum neutron flux and a velocity of about 800 m/s, with a wavelength spread of ∆λ/λ = 10%, and a collimation distance of 4 m. A resultant flux of about 5 × 10^6^ neutrons/s is achieved at the sample position. The sample, embedded by sapphire cover slips is placed at the goniometer center and neutrons vertically pass through it. It is important to note that the sapphire coverslips, used for embedding the brain section are transparent to neutrons. A 2D ^6^Li scintillation detector is placed behind the sample at a distance of 370 mm, in parallel to the sample plane to cover the required *q*-range. The flight path between the sample and detector was shielded with a vacuum tube to reduce air-scattering and gather the scattering patterns from the sample. The blue square spot at the center of each image corresponds to the beam-stopper for blocking the un-scattered (directly transmitted) beam and to protect the detector sensor.

To investigate the structure of the brain section, here, we used the collimated neutron beam, which has a small wavelength (5 Å), narrow wavelength distribution, and low absorbance. Using this setting, the scattering patterns were collected in transmission geometry with a detector of area approx. 3500 cm^2^. The detector contains thousands of sensors of size 5.3 mm. Neutrons scatter coherently from the myelin sheaths present in the brain section. The scattering patterns were acquired for an exposure time of 30 min at each scanning position, such as exemplarily shown in Fig. 2 with sufficiently high signal-to-noise ratio. The sample was scanned at a spatial resolution of 1 mm, moving the sample holder in steps of 1 mm horizontally or vertically after each exposure.  During the neutron scattering measurement (Fig. 7), the sample had an anti-clockwise in-plane rotation offset of 35° between the interhemispheric fissure and the vertical laboratory reference. At first, a coarse transmission scan was carried out using the total intensity of the beam to define the sample position in the circular hole from an absorption map (Fig. S8). The uniformity of the sample-thickness (60 μm) was cross-checked by the normalized transmission of neutrons through the section (Fig. S9) thereafter followed by the raster scan to map the scattering patterns of the entire brain section. While scanning, 10 positions inside the tissue and several positions outside the sample were skipped due to time constraints. For rest of the sample, the SANS measurements took about 2 days (48 hours).

### SANS data analysis

Data processing of the scanning SANS measurements was performed using in-house software. The scanning over the sample leads to an array of 2D scattering patterns. The scattering patterns from a model fiber (having oriented myelin sheaths across a cylindrical axon) at different inclination angles with respect to neutron propagation have been simulated for comparison. The detail of the modelling is available in the Supplementary Information^39^. Each of the scattering pattern corresponds to a local average of the scattering signal over the illuminated area (1 mm × 1 mm). In order to have consistency among all measurements, each pattern was normalized to the incident beam flux. A scattering pattern from an empty part of the circular window (acts as background) was subtracted from each pattern of the sample. The radial intensity profiles as a function of the momentum transfer vector (*q*) are extracted from the 2D scattering patterns by integrating azimuthally over the intensity of each pixel. The contrast maps at the sample plane were computed from each scattering pattern. Depending upon the signal level, the scattering contributions were considered for two different *q*-regimes, namely, Δ*q*_*Porod*_: 0.019–0.048 Å^−1^ (close to the center of the beam) and Δ*q*_*myelin*_: 0.07–0.098 Å^−1^ (across the myelin Bragg peak). The scattering contribution for each regime was integrated over the corresponding *q* range and mapped separately for every scanning position. The distribution of the integrated scattering intensity in the Porod regime (Δ*q*_*Porod*_) results in the dark-field map^58^. This map provides an estimation of large microstructures over the sample at low angles, whereas the intensity maps of the myelin diffraction peak regime (Δ*q*_*myelin*_) give an estimation of myelin present across the section. Here, we lay stress on the model free analysis of the entire data, while the choice of q-ranges depends on the literature-based experience^41^.

The anisotropy of the SANS pattern gives insight to the orientation of the brain structure on the nanoscale^59^. To determine the intensity profile in the Porod and Bragg regimes, each 2D scattering pattern was divided into 18 azimuthal segments, and then each segment was integrated radially i.e. as a function of *q*^35,60^. The scattering intensity values of the diagonal segments are weighted averages over the corresponding number of covered detector sensors to achieve the inversion symmetry of the pattern, resulting from the analysis of 9 segments.

To determine the multiplicity of fiber orientations in each scanning position in the Bragg regime the intensity of the myelin scattering on the DS arcs was integrated radially within a constant *q*-value window (Δ*q*_*myelin*_) over an azimuth segment of 20° to create a set of 18 vertices for a full circle scattering intensity profile (Fig. 4B). A simple peak finder was applied to extract the Bragg peaks from each profile. The fiber directions where calculated adding +90° and -90° respectively to the Bragg peak azimuth values. The prominence of each peak was calculated dividing the peak height by the total signal amplitude of the 360° profile. Finally the bilateral peak width was determined from the range to both neighboring local minima. The same procedure was applied to calculate the profiles in the Porod regime however at a lower *q*-value range (Δ*q*_*Porod*_).

### 3D-PLI measurement

The 3D-PLI technique reveals the spatial pathways of nerve fibers with a resolution of a few micrometers, allowing to explore fiber bundles and even individual cortical fibers utilizing their birefringence. The measurement was carried out with the polarimeter LMP-1 from Taorad GmbH, Aachen, using a numerical aperture of 0.15 and an object-space resolution of 1.3 μm/pixel. Unpolarized light from a single white LED filtered by a narrow-band filter (550 nm) passes a rotatable polarizer. The light interacts with the brain tissue mounted on a movable horizontal stage. A polarization filter and a quarter-wave retarder mounted with a principal axis offset of 45° with respect to the polarizer plane serves as circular analyzer. The transmitted light is captured by a CCD camera (Retiga 4000R, QImaging, Canada) with 2048 × 2048 pixels at a pixel size of 1.3 × 1.3 μm^2^. The analyzer is rotated in steps of 20°, yielding a series of nine images per tile. Each tile has a rectangular field of view with a border length of 2.7 mm and 28% overlap with the neighboring tiles. The whole sample has been covered by 4 × 6 tiles during the measurement.

### 3D-PLI data analysis

The transmittance measured in 3D-PLI microscopy depends on the absorption of the polarized light by the brain tissue as well as on the scattering of light by its constituents. The absorption of the light by the brain tissue is high, and light scattering is incoherent and destructive (light with large wavelength and finite wavelength distribution) when it passes through the brain section. Moreover, as the different parts of the section consist of different tissue matters, the light scatters differently through a complex light-tissue interaction. Therefore, the transmitted light undergoes multiple scattering events interacting with tissue at the microscopic level.

The measured intensity profiles of each pixel over the nine images were analyzed to extract the spatial distribution and orientations of the nerve fibers. The light intensity of each pixel varies during rotation of the analyzer in a sinusoidal manner:

1 $$I\left( {\rho ,\varphi ,\delta } \right) = \frac{{I_{T} }}{2}\left( {1 + {\text{sin}}\left( {2\left( {\rho - \varphi } \right)} \right)\sin \delta } \right),$$

where $\rho$, $\varphi$, $\frac{{I_{T} }}{2}$, $\sin \delta$ are the analyzer plane angle, the in-plane fiber orientation angle, the transmittance and the retardation respectively. The global transmittance of the signal is obtained by averaging the transmitted light intensities measured. It’s important to note that conventional transmission microscopy with unpolarized light yields the same result for the global transmission. The resultant transmittance values are normalized by the average light transmittance without sample.

The retardation ($\sin \delta$) of the signal depends on the birefringence of the fibers ($\Delta n$), the section thickness (t_s_) and the wavelength of illumination ($\lambda$). It corresponds to the fiber inclination angle ($\alpha$) i.e. the fiber elevation out of the section plane via the relation

2 $$\delta = \frac{{{\uppi }}}{2}{\text{t}}_{{{\text{rel}}}} {\text{cos}}^{2} \left( \alpha \right),$$

where t_rel_ is the relative thickness of the section and ${\text{t}}_{{{\text{rel}}}} = 4\frac{{{\text{t}}_{{\text{s}}} \Delta {\text{n}}}}{{{\uplambda }}}$.

Hence 3D-PLI yields the predominant fiber orientation directly from the measured data by evaluating the phase and the amplitude of the intensity profile of each pixel sampled during the rotation of the analyzer.

### Registration of sSANS and 3D-PLI data

SANS and 3D-PLI measurements were carried out on a single sample but using two different mounting platforms as sample holders. The sample mounting for two different measurements is unlikely to be collinear with each other. Therefore, one needs to perform a registration of two different images from two different measurements to show the correspondences. Hence, we registered the 3D-PLI images measured beforehand to the 11 × 11 mm^2^ beam grid of the SANS measurement by a rigid affine transformation, yielding 8460 × 8460 pixels in order to overlay the SANS results (fiber orientation bars) directly to the 3D-PLI reference with native resolution (1.3 μm pixel size). Afterwards the registered 3D-PLI vector array was interpolated on each square millimeter grid position to compare the SANS results with the average fiber orientations of 3D-PLI on each tile individually. We looked for the best possible fit in terms of symmetry and intensity distribution by rotating and translating the peripheral transmission image. We registered the 3D-PLI transmittance on top of the SANS maps by employing an orientational and translational off-set of about 35° and 0.4 mm along the negative y-axis (Figs. 1, 2, 3). The FOM calculated from the 3D-PLI data has a spatial resolution of 1.3 μm (Fig. 1). We estimated the 3D-orientation of the fiber architecture at 1 mm resolution integrating 769 × 769 3D-PLI vectors into a single spherical histogram to compare the results from the two different techniques.

## Supplementary Information

Supplementary Information 1.

Supplementary Video 1.
