# Supplementary material for: Distribution and orientation of nerve fibers and myelin assembly in a brain section retrieved by small-angle neutron scattering
Source: Sci Rep. 2021 Aug 27;11:17306. doi: 10.1038/s41598-021-92995-2 (PMC8397781; doi:10.1038/s41598-021-92995-2)
Supplement: Supplementary file 1 — Supplementary Information 1. [file 41598_2021_92995_MOESM1_ESM.docx]

Supplementary Information

Distribution and Orientation of Nerve Fibers and Myelin Assembly in a Brain Section Retrieved by Small-Angle Neutron Scattering

S. Maiti^1,2^, H. Frielinghaus^3^, D. Gräßel^2^, M. Dulle^1^, M. Axer^2^, S. Förster^1,4,*^

^1^Jülich Centre of Neutron Science (JCNS-1/IBI-8), Forschungszentrum Jülich GmbH, 52425 Jülich, Germany

^2^Institute of Neuroscience and Medicine (INM-1), Forschungszentrum Jülich GmbH, 52425 Jülich, Germany

^3^Jülich Centre for Neutron Science at Heinz Maier-Leibnitz Zentrum (JCNS-MLZ), Forschungszentrum Jülich GmbH, 85748 Garching, Germany

^4^ Institute of Physical Chemistry, RWTH Aachen University, 52074 Aachen, Germany

1. Animation of scattering patterns and the extracted parameters

See video.

2. Scattering patterns of oriented myelin structures


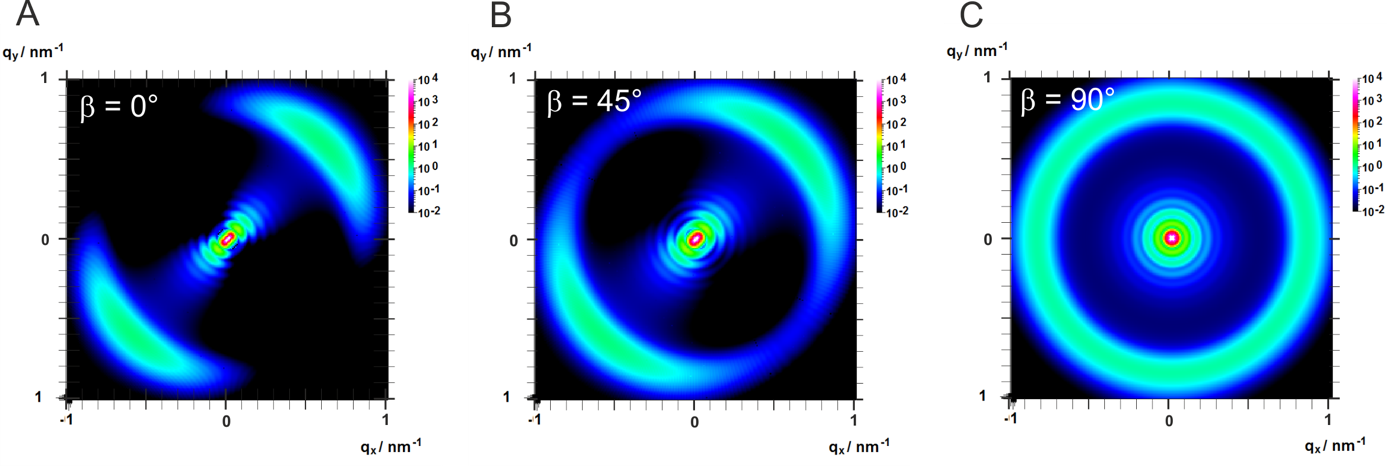


**Figure S1:** Calculated scattering patterns for cylindrically-shaped axons surrounded by a myelin sheath, where the axon orientation is in-plane, corresponding to an inclination angle of 0° (A), has an inclination angle of 45° (B), and an inclination angle of 90°, corresponding to a vertical orientation. The primary beam direction is normal to the q_x_/q_y_-scattering plane.

To investigate the effect of axon inclination on the anisotropy of the scattering pattern, we performed model calculations, where the axon was approximated by a core/shell-cylinder of length L and core radius Rc, inclined by an angle β with respect to the scattering plane, and surrounded by a lipid membrane multilayer with periodic spacing l_layer_. Details of the model are shown in Figure S2.


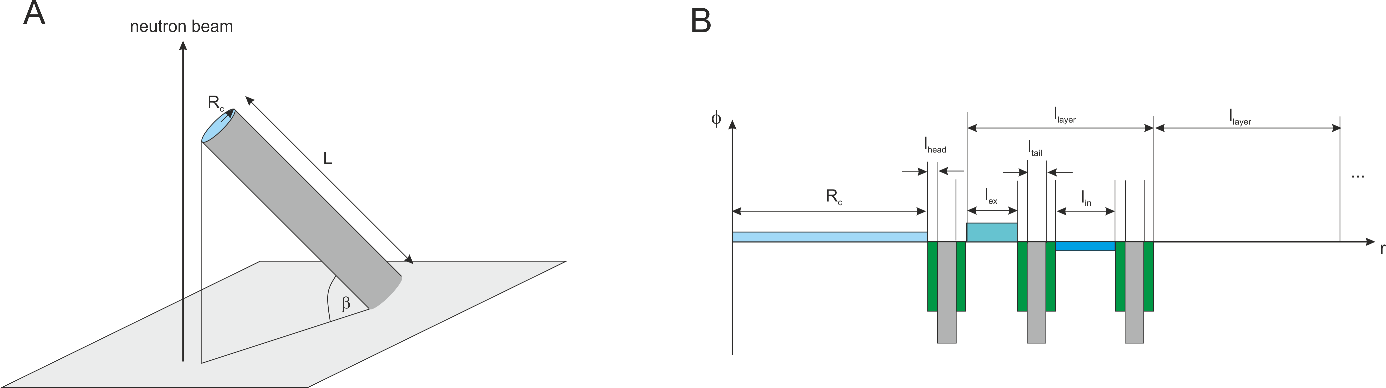


**Figure S2:** A) Orientation of the cylinder of length L and cross-sectional radius R_c_ with respect to the neutron beam and the scattering plane, with the inclination angle β. B) Cross-section of the lipid multilayer representing the myelin sheath consisting of a number of periodically spaced lipid bilayers. Indicated are the cross-sectional diameters of the lipid head group l_head_, lipid tail group l_tail_, intracellular diameter l_in_, extracellular spacing l_ex_, and the periodicity l_layer_ comprising two bilayers making up a cell. The myelin sheath consists of a number ‘*n’* of these cells. The scattering length density (φ) of the individual sections as a function of radius (r) are indicated by different colors.

The scattering patterns were calculated for L = 5000 nm, R_c_ = 500 nm, l_head_ = 1.0 nm, l_tail_ = 2.0 nm, l_ex_ = l_in_ = 3.5 nm, n = 6, f_c_ = f_ex_ = f_in_ = 0.001, f_head_ = -0.55, f_tail_ = -0.8, and a Gaussian orientational distribution function around the main cylinder direction corresponding to an orientational order parameter of S = 0.83. For an inclination angle of β = 0° we observe wto distinct arcs with a pronounced low-q-scattering centered on the connecting line between the two arcs. For an inclination angle of β = 45° we observe an azimuthal broadening of the arcs, located on top a Debye-Scherrer ring. For an inclination angle of β = 90° we observe the expected isotropic scattering pattern with a fully developed Debye-Scherrer ring. From comparison to the experimental scattering pattern in Figures 2 and in supplementary video we conclude from the presence of a faint Debye-Scherrer ring, that the experimental inclination angle is not zero, but small, i.e. much smaller than 45°.

[1] S. Förster, S. Fischer, K. Zielske, C. Schellbach, M. Sztucki, P. Lindner, J. Perlich, Calculation of scattering patterns of ordered nano- and mesoscale materials, Adv. Coll. Int. Sci 163 (2011) 53-83.


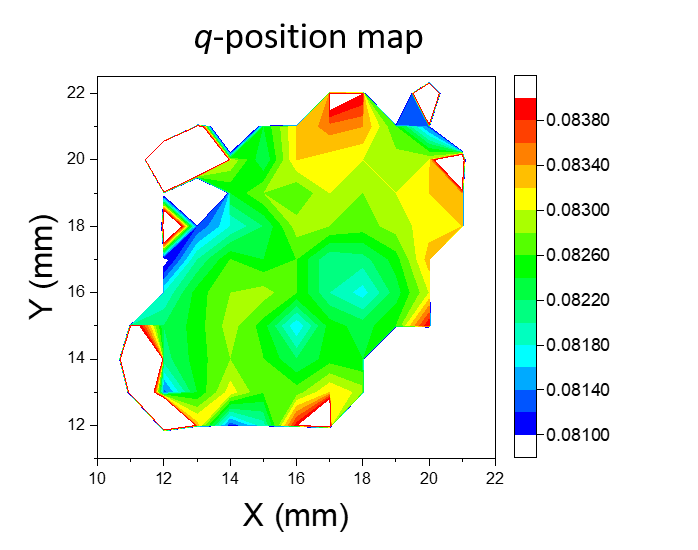


**Figure S3:** Map of the myelin Bragg peak position (q_max_) to estimate the thickness distribution over the entire section.


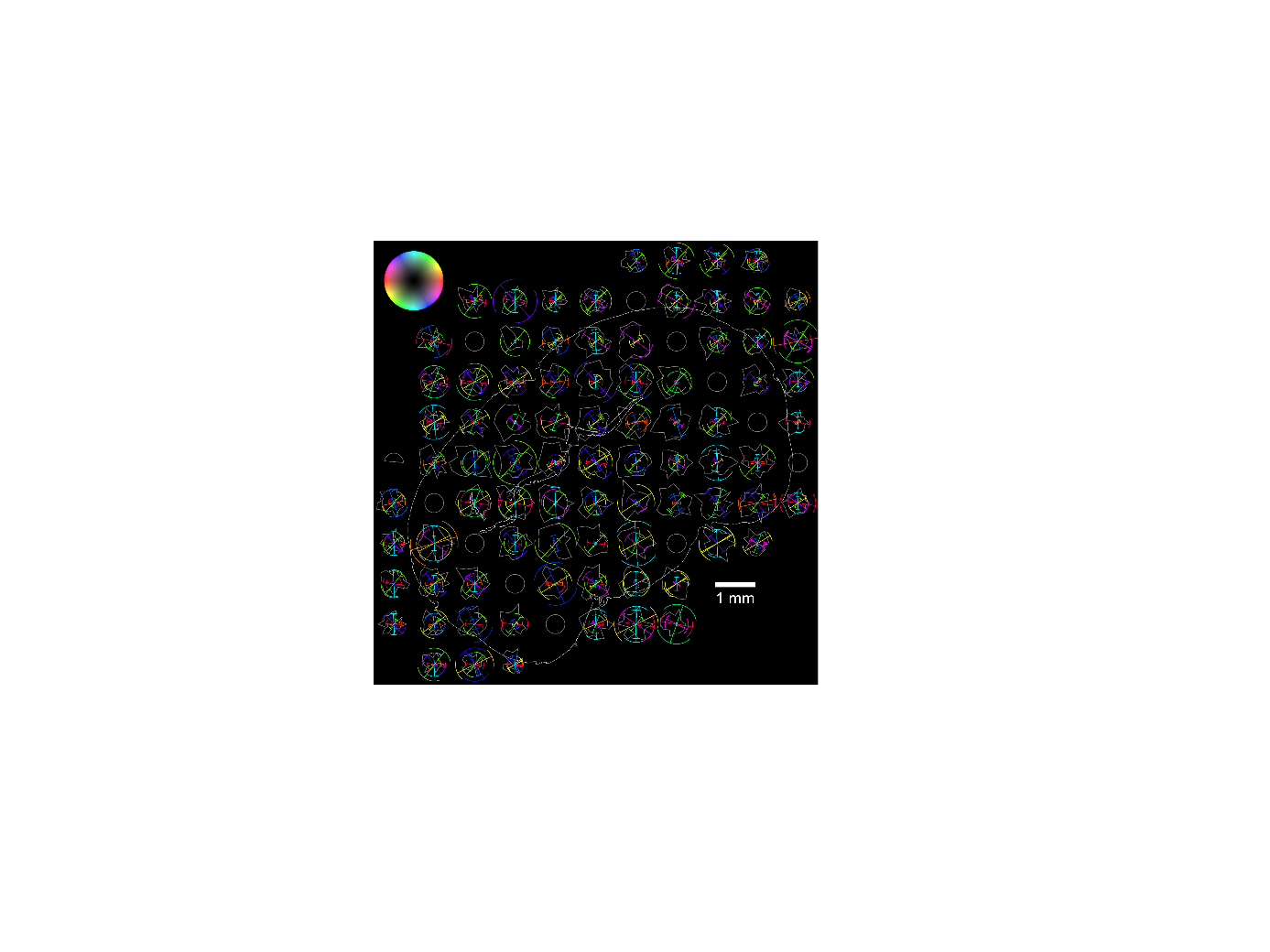


**Figure S4:** Bragg peak intensity profile and orientation map of the fibers including the peak width

**
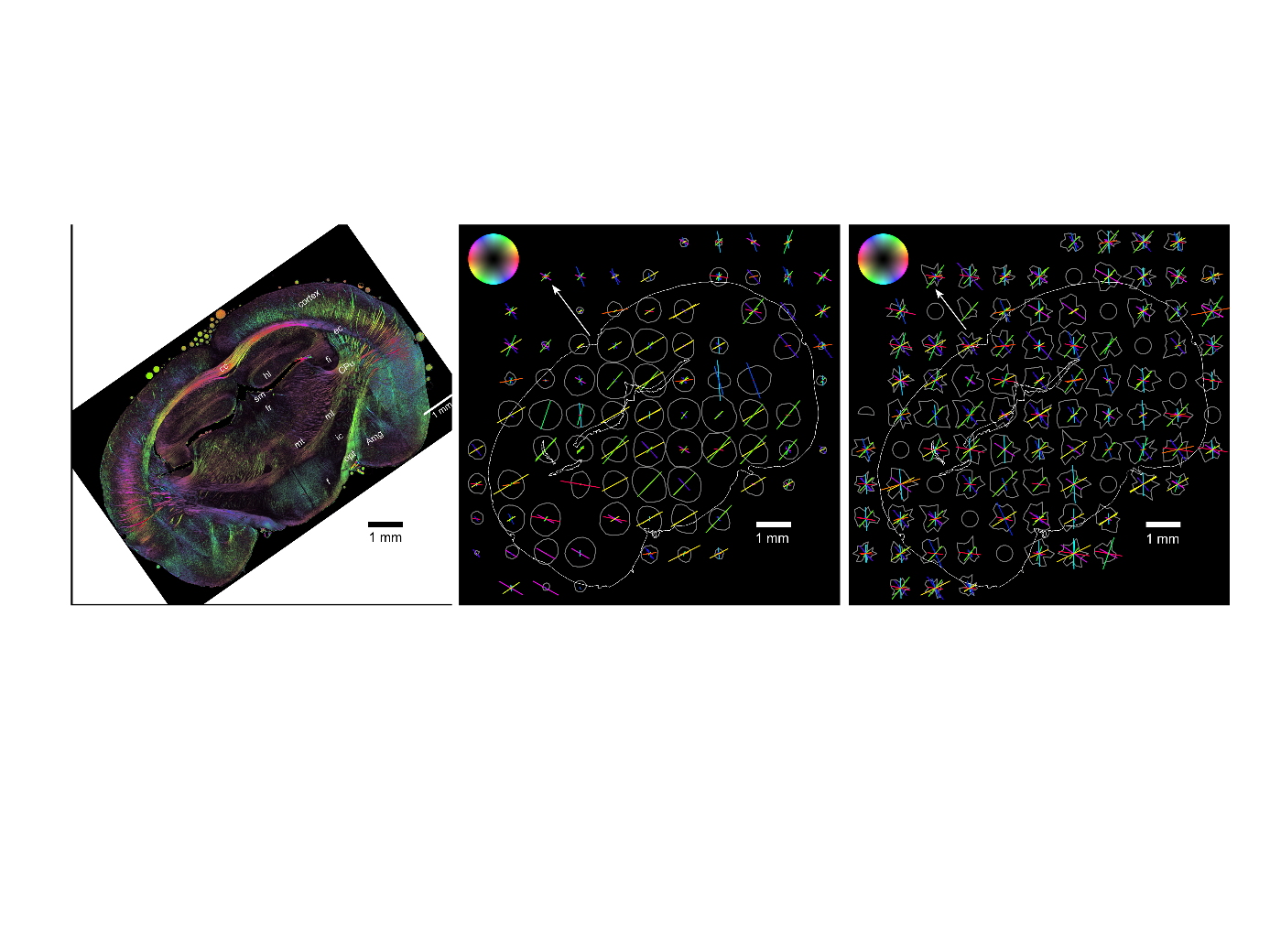
**

**Figure S5:** Comparison of the histology (Fig. 1) and SANS (Figs. 3B, 4B)

**
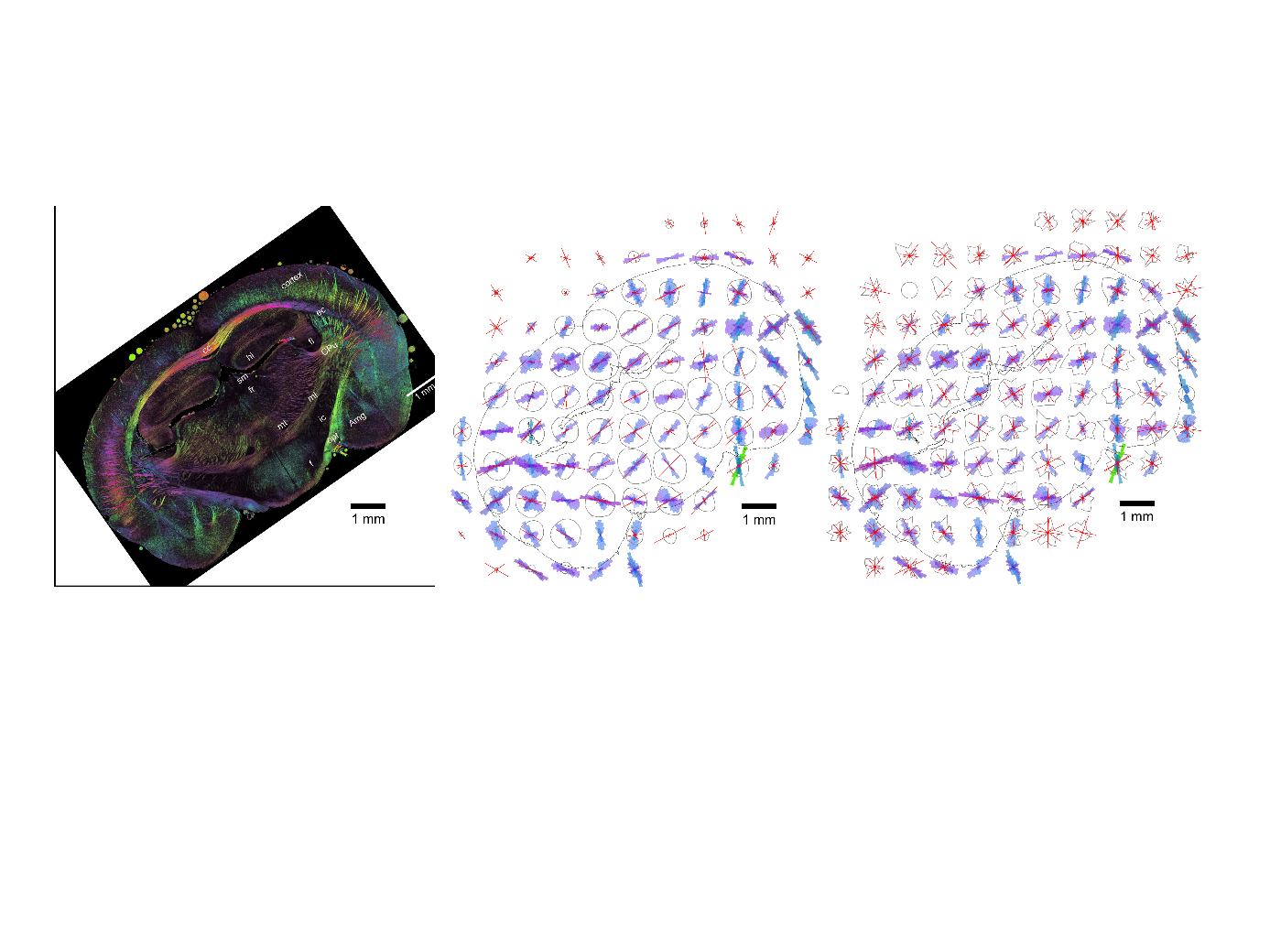
**

**Figure S6:** Comparison of the histology (Fig. 1), SANS and PLI (Figs. 6C and 6D)


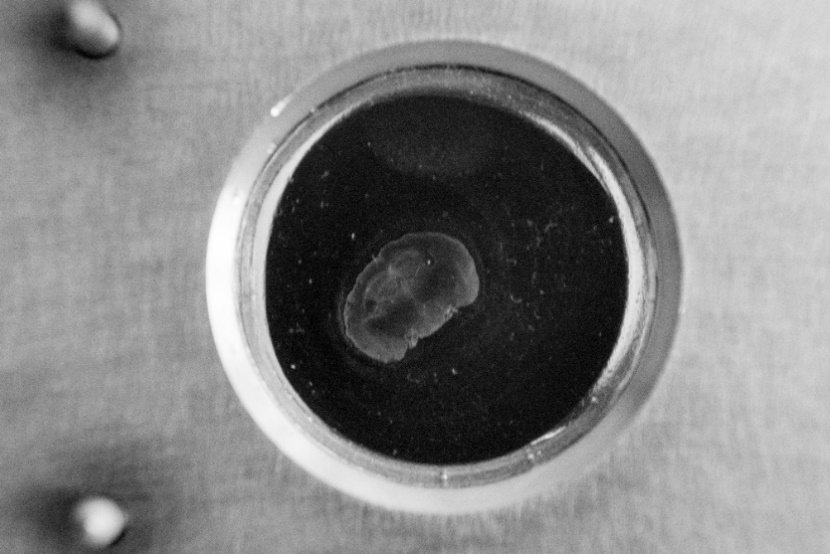


**Figure S7:** Photographic image of mouse brain section embedded between two sapphire windows of the sample holder.


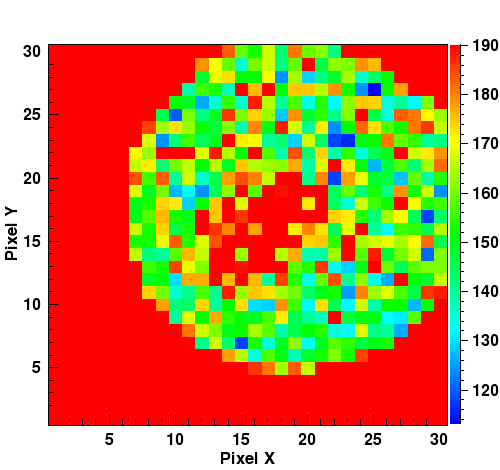


**Figure S8:** Absorption map (pixel) of the transmitted intensity of a scan across the sapphire window shown in Figure S7 to identify the brain section position. The red area marked by the arrows shows the location of the brain section.


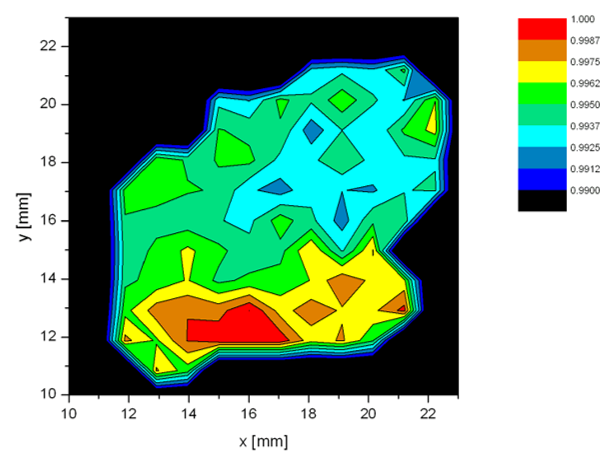


**Figure S9:** Normalized intensity map of the transmitted SANS scans from the brain section to determine the uniformity of the thickness over the whole section.
